# Factorized Duality, Stationary Product Measures and Generating Functions

**DOI:** 10.1007/s10955-018-2090-1

**Published:** 2018-06-22

**Authors:** Frank Redig, Federico Sau

**Affiliations:** Delft Institute of Applied Mathematics, Delft University of Technology, Mekelweg 4, 2628 CD Delft, The Netherlands

**Keywords:** Duality, Generating function, Intertwining, Interacting particle systems, Orthogonal polynomials

## Abstract

We find all self-duality functions of the form

$$\begin{aligned} D(\xi , \eta )= \prod _{x} d(\xi _x, \eta _x) \end{aligned}$$

for a class of interacting particle systems. We call these duality functions of simple factorized form. The functions we recover are self-duality functions for interacting particle systems such as zero-range processes, symmetric inclusion and exclusion processes, as well as duality and self-duality functions for their continuous counterparts. The approach is based on, firstly, a general relation between factorized duality functions and stationary product measures and, secondly, an intertwining relation provided by generating functions. For the interacting particle systems, these self-duality and duality functions turn out to be generalizations of those previously obtained in Giardinà et al. (J Stat Phys 135:25–55, 2009) and, more recently, in Franceschini and Giardinà (Preprint, arXiv:1701.09115, 2016) . Thus, we discover that only these two families of dualities cover all possible cases. Moreover, the same method discloses all simple factorized self-duality functions for interacting diffusion systems such as the Brownian energy process, where both the process and its dual are in continuous variables.

## Introduction

Duality and self-duality are very useful and powerful tools that allow to analyze properties of a complicated system in terms of a simpler one. In case of self-duality for particle systems, the dual system is the same and the simplification arises because in the dual one considers only a finite number of particles (e.g. [3]).

Several methods are available to construct dual processes and duality relations. In the context of population dynamics, the starting point to find dualities is to consider the coalescent, the simplest example here being the duality between Kingman’s coalescent block-counting process and the Wright-Fisher diffusion (for an overview of this kind of dualities in more general contexts, see [2]). Another method is provided by the pathwise dualities based on graphical constructions and time reversal, see e.g. [15, 19].

In the context of conservative particle systems such as the exclusion process and its generalizations, zero range processes, etc. (cf. [3, 15]), the algebraic method developed in [9] offers a general framework to construct self-duality functions starting from a reversible product measure by using symmetries of the generator, i.e. operators commuting with the generator. Additionally, if these symmetries are in product form, i.e. of the form $\prod _{x} S_x$, where the action of $S_x$ depends only on the variables associated to site *x*, then the self-duality functions produced by these product-like symmetries and a reversible product measure also *factorize over the sites*, i.e. are a product over the sites of functions that depend only on the variables associated to that site. In this paper, we call such duality functions “*simple factorized self-duality functions*”.

A complete picture of how to obtain *all* simple factorized self-duality functions for such particle systems is missing (except in the simplest case of symmetric simple exclusion, see e.g. [18] and references therein). Natural associated questions are: which of these conservative particle systems allow self-duality and is it possible then to obtain all simple factorized self-duality functions for these systems? One of the useful applications of disposing of all simple factorized self-duality functions is that, depending on the target, one can choose appropriate ones: e.g. in the hydrodynamic limit and the study of the structure of the stationary measures, the “classical” duality functions are the appropriate ones (see e.g. [3]), whereas in the study of (stationary and non-stationary) fluctuation fields and associated Boltzmann-Gibbs principles [12], Chap. 11], as well as in the study of speed of relaxation to equilibrium in $L^2$ or in the study of perturbation theory around models with duality (cf. [3]), “orthogonal” duality functions turn out to be very useful.

In this paper, we develop an approach to answer the above questions and systematically determine all simple factorized self-duality functions for a class of conservative interacting particle systems. As a consequence, by considering many-particle limits, we also obtain simple factorized dualities and self-dualities for a class of conservative diffusion processes, such as the Brownian energy process.

In this route, starting from examples, we first investigate a general connection between stationary product measures and simple factorized duality functions. This shows, in particular, that for infinite systems with simple factorized self-duality functions, the only stationary measures which are ergodic (w.r.t. either space-translation or time) are in fact product measures. Then we use this connection between stationary product measures and simple factorized duality functions to recover all possible candidate simple factorized duality functions from the stationary product measures. More precisely, we show that, given the first duality function, i.e. the duality function with a single dual particle, all other simple factorized duality functions are determined. This provides a simple machinery to obtain all simple factorized self-duality functions in processes such as Symmetric Exclusion Process ($\text {SEP}$), Symmetric Inclusion Process ($\text {SIP}$) and Independent Random Walkers ($\text {IRW}$). In particular, we recover via this method all orthogonal polynomial duality functions obtained in [8].

Moreover, we prove that in the context of conservative particle systems where the rates for particle hopping depend only on the number of particles of the departure and arrival sites, the processes $\text {SEP}, \text {SIP}$ and $\text {IRW}$ are the only systems which have self-duality with simple factorized self-duality functions and that the first duality function is necessarily an affine function of the number of particles. Next, in order to prove that the only candidate simple factorized self-duality functions derived via the method described above are actual self-duality functions, we develop a method based on generating functions. This method, via an intertwining relation, allows to go from discrete systems (particle hopping dynamics) to continuous systems (such as diffusion processes or deterministic dynamics) and back, and also allows to pass from self-duality to duality and back. The proof of a self-duality relation in a discrete system then reduces to the same property in a continuous system, which is much easier to check directly. The generating function method also provides new examples of self-duality for processes in the continuum such as the Brownian Energy Process ($\text {BEP}$), which intertwines with the $\text {SIP}$ via the generating function. In fact, we show equivalence between self-duality of $\text {SIP}$, duality between $\text {SIP}$ and $\text {BEP}$ and self-duality of $\text {BEP}$. Finally, this method based on generating functions generalizes the concept of obtaining dualities from symmetries to intertwinings, being a symmetry an intertwining of the generator with itself.

Notice that we restrict in this paper to conservative particle systems (and associated diffusion processes) in *discrete space*, although there are many other possible contexts such as spin-systems, reaction diffusion systems, Fleming–Viot processes, etc. as well as continuum-space limits of such systems where similar duality questions could be addressed. Moreover, most of the emphasis will be on self-duality, as dualites in this context in fact follow from self-dualities.

**Organization of the Rest of the Paper** In Sect. 2 we introduce the basic definitions of duality and systems considered. Additionally, in Theorem 2.1 we prove which particle systems out of those considered admit simple factorized self-duality. In Sect. 3, we investigate a general relation between simple factorized duality functions and stationary product measures. We treat separately the finite and infinite contexts in which this relation arises; in the latter case, we exploit this connection to draw some conclusions on the product structure of ergodic measures. Section 4 is devoted to the derivation of all possible simple factorized self-duality and duality functions. Here Theorem 2.1 and the relation in the previous section are the two key ingredients. In Sect. 5, after an introductory example and a brief introduction on the general connection between duality and intertwining relations, we establish an intertwining between the discrete and the continuum processes. This intertwining relation is then used to produce all the self-duality functions for the Brownian Energy Process.

## Setting

We start defining what we mean by *duality* for Markov processes. Then, we introduce a general class of Markov interacting particle systems with associated interacting diffusion systems arising as many-particle limits.

**Main Result of the Section** Theorem 2.1 states that the only conservative particle systems described by the infinitesimal generator (7) below which admit a “non-trivial” factorized self-duality are necessarily $\text {IRW}, \text {SIP}$ and $\text {SEP}$-type of processes. Moreover, in the same statement, we find the general form of the first single-site self-duality function for such systems.

### Duality

Given two (Polish) state spaces $\Omega$ and ${\hat{\Omega }}$ and *two Markov processes* $\{\xi (t), t\ge 0\}$ and $\{\eta (t),\ t\ge 0\}$ evolving on them, we say that they are *dual* with duality function $D: {\hat{\Omega }} \times \Omega \rightarrow {\mathbb {R}}$ (where *D* is a measurable function) if, for all $t>0, \xi \in {\hat{\Omega }}$ and $\eta \in \Omega$, we have the so-called duality relation

1 $$\begin{aligned} {\hat{{\mathbb {E}}}}_\xi D(\xi (t),\eta )= {\mathbb {E}}_\eta D(\xi ,\eta (t)). \end{aligned}$$

If the laws of the two processes coincide, we speak about *self-duality*.

More generally, we say that *two semigroups* $\{S(t), t \ge 0\}$ and $\{ {\hat{S}}(t), t\ge 0\}$ are dual with duality function *D* if, for all $t \ge 0$,

2 $$\begin{aligned} ({\hat{S}}(t))_{} D= (S(t))_{\mathrm{right}}D, \end{aligned}$$

where “left” (resp. “right”) refers to action on the left (resp. right) variable. In the case that these semigroups are Markov semigroups, (1) is exactly the same as (2).

Even more generally, we say that *two operators* *L* and ${\hat{L}}$ are dual to each other with duality function *D* if

3 $$\begin{aligned} ({\hat{L}})_{} D= (L)_{\mathrm{right}}D. \end{aligned}$$

In the context of Markov processes, the operators *L* and ${\hat{L}}$ which we have in mind here are the generators of the Markov processes. Moreover, we refer to [11] for more technical aspects of duality relations, e.g. when generator duality implies semigroup duality or which are the exact restrictions on the state spaces needed.

In order not to overload notation, we use the expression $A_{} D(\xi ,\eta )$ for $(A D(\cdot , \eta ))(\xi )$ and, similarly, $B_{\mathrm{right}} D(\xi ,\eta )= (BD(\xi ,\cdot ))(\eta )$. We will often write $D(\xi ,\eta )$ in place of $(\xi ,\eta )\mapsto D(\xi ,\eta )$.

### Lattice and Factorization over Sites

The underlying geometry of all systems that we will look at consists of a set of sites *V* either finite or $V={\mathbb {Z}}^d$. Moreover we are given a family of transition rates $p: V \times V \rightarrow {\mathbb {R}}_+$, satisfying the following conditions: for all $x, y \in V$,

1. *Vanishing diagonal* $$ p(x,x)=0,$$
2. *Irreducibility* there exist $x_1=x, x_2, \ldots , x_m=y$ such that $\prod _{l=1}^{m-1} p(x_l, x_{l+1} ) > 0.$

In case of infinite *V*, we further require the following:

(3) *Finite-range* there exists $R> 0$ such that, for all $x,y \in V, p(x,y)=0$ if $|x-y|>R,$
(4) *Uniform bound on total jump rate* $\sup _{x \in V} \sum _{y \in V} p(x,y) < \infty .$

Notice that when *p* is finite-range and translation invariant, then the uniform bound on total jump rate follows automatically. Notice also that the finite-range assumption is not necessary and can be relaxed; however, we assume it here for simplicity in order to avoid existence problems of the processes in the context when $V={\mathbb {Z}}^d$.

To each site $x \in V$ we associate a variable $\eta _x \in E= {\mathbb {N}}$, $\{0,\ldots ,N\}$ or ${\mathbb {R}}_+$, with the interpretation of either the number of particles or the amount of energy associated to the site *x*. Configurations are denoted by $\eta \in \Omega =E^V$.

As discussed in the introduction, we look at duality functions which *factorize over sites*, i.e. of the form

4 $$\begin{aligned} D(\xi ,\eta )= \prod _{x\in V} d(\xi _x,\eta _x),\quad \eta \in E^V,\quad \xi \in {{\hat{E}}}^V. \end{aligned}$$

We then call the functions $d(\xi _x,\eta _x)$ the *singe-site duality functions* and further assume

5 $$\begin{aligned} d(0,\cdot )\equiv 1. \end{aligned}$$

The above condition (5) is related to the fact that we want to have duality functions which make sense for infinite systems when the dual configuration has a finite total mass. A typical example is when $\eta , \xi \in {\mathbb {N}}^{{\mathbb {Z}}^d}$, where $\eta$ is an infinite configuration while $\xi$ is a finite configuration, so that in the product (4) there are only a finite number of factors different from $d(0, \eta _x)$. In this sense, the choice $d(0,\cdot )\equiv 1$ is the only sensible one for infinite systems. When *V* is finite and $E = {\mathbb {N}}$ or $\{0,\ldots ,N \}$, this condition is not necessary and e.g. if a (positive) reversible product measure $\mu = \otimes _{x \in V} \nu$ exists, then the so-called *cheap self-duality function* $D(\xi ,\eta )=\tfrac{1}{\mu (\xi )} {\mathbb {1}}{\{\xi =\eta \}} = \prod _{x \in V} \frac{1}{\nu (\xi _x)} {\mathbb {1}}\{\xi _x = \eta _x \}$ does *not* satisfy (5).

### Interacting Particle Systems with Simple Factorized Self-duality

The class of interacting particle systems we consider is described by the (formal) infinitesimal generator acting on local functions $f : \Omega \rightarrow {\mathbb {R}}$ as follows:

6 $$\begin{aligned} L f(\eta )= & {} \sum _{x,y \in V} p(x,y) L_{x,y} f(\eta ),\quad \eta \in \Omega , \end{aligned}$$

where $L_{x,y}$, the *single-edge generator*, is defined as

7 $$\begin{aligned} L_{x,y}f(\eta )= & {} u(\eta _x) v(\eta _y) (f(\eta ^{x,y})-f(\eta ))\nonumber \\&+u(\eta _y)v(\eta _x)(f(\eta ^{y,x})-f(\eta )),\quad \eta \in \Omega , \end{aligned}$$

and $\eta ^{x, y}$ denotes the configuration arising from $\eta$ by removing one particle at *x* and putting it at *y*, i.e. $\eta ^{x,y}_x= \eta _x-1, \eta ^{x,y}_y= \eta _y+1$, while $\eta ^{x,y}_z= \eta _z$ if $z \ne x,y$. Note the conservative nature of the system and the form of the particle jump rates in (7) which depend on the number of particles in the departure and arrival site in a factorized form. Minimal requirements on the functions *u* and *v*, namely

(i) $u(0)=0, u(1)=1$ and $u(n) > 0$ for all $n > 0,$
(ii) $$v(0)\ne 0$$ and $$v(N)=0$$ if $$E=\{0,\ldots ,N \}$$ and in all other cases $$v(n)>0 $$,

guarantee the existence of a one-parameter family of *stationary* (actually *reversible*) *product measures* $\{\otimes \nu _\lambda , \lambda > 0 \}$ with marginals $\nu _\lambda$ given by

8 $$\begin{aligned} \nu _\lambda (n)= & {} \varphi (n) \frac{\lambda ^n}{n!} \frac{1}{Z_\lambda },\quad n \in {\mathbb {N}}\;\text {or}\;\{0,\ldots ,N\}, \end{aligned}$$

for all $\lambda > 0$ for which the normalizing constant $Z_\lambda < \infty$ and with $\varphi (n)=n! \prod _{m=1}^{n}\frac{v(m-1)}{u(m)}$.

#### Remark 2.1

The existence of the processes with formal generator (6) poses no problem if *V* is finite. If *V* is infinite, further growth conditions on the functions *u*(*n*) and *v*(*n*) are required in order to ensure non-explosion. For the processes that we will be considering in the next sections, which have the self-duality property, existence can be proved via self-duality as in [3], Sect. 2.2.4].

### Basic Examples and Characterization of Self-dual Particle Systems

We recall here the basic examples of self-dual interacting particle systems and corresponding simple factorized self-duality functions known in literature (cf. e.g. [9]). Next, in Theorem 2.1 below we prove that these are the only particle systems (within our defined class) that are self-dual with simple factorized self-duality functions.

(I) *Independent random walkers* ($\text {IRW}$)
$$E={\mathbb {N}},$$
$$u(n)=n, v(n)=1,$$
$$\nu _\lambda \sim \text {Poisson}(\lambda ),$$ $$\nu _\lambda (n)=\tfrac{\lambda ^n}{n!} e^{-\lambda }, \quad \lambda > 0,$$
$$d(k,n)= \frac{n!}{(n-k)!} {\mathbb {1}}\{k \le n \}.$$
(II) *Symmetric inclusion process* ($\text {SIP}(\alpha ), \alpha > 0$)
$$E={\mathbb {N}},$$
$$u(n)=n, v(n)=\alpha +n,$$
$$\nu _\lambda \sim \text {Gamma}_{\text {d}}(\alpha ,\lambda ), \nu _\lambda (n)= \tfrac{\Gamma (\alpha +n)}{\Gamma (\alpha )} \tfrac{\lambda ^n}{n!}(1-\lambda )^\alpha ,\; \lambda \in (0,1),$$
$$d(k,n)= \tfrac{\Gamma (\alpha )}{\Gamma (\alpha +k)}\tfrac{n!}{(n-k)!} {\mathbb {1}}\{k \le n \}.$$
(III) *Symmetric exclusion process* ($\text {SEP}(\gamma ), \gamma \in {\mathbb {N}}$)
$$E=\{0,\ldots ,N \},$$
$$u(n)=n, v(n)=\gamma -n,$$
$$\nu _\lambda \sim \text {Binomial}(\gamma ,\tfrac{\lambda }{1+\lambda }), \nu _\lambda (n)= \tfrac{\gamma !}{(\gamma -n)!}\tfrac{\lambda ^n}{n!} \left( \tfrac{1}{1+\lambda }\right) ^\gamma ,\; \lambda >0,$$
$$d(k,n)= \tfrac{(\gamma -k)!}{\gamma !}\tfrac{n!}{(n-k)!} {\mathbb {1}}\{k \le n \}.$$

In the following theorem we show that the only processes with generator of the type (6) which have non-trivial simple factorized self-duality functions are of one of the types described in the examples above, i.e. $\text {IRW}, \text {SIP}\ or\ \text {SEP}$. Here by “non-trivial” we mean that the first single-site self-duality function *d*(1, *n*) is not a constant (as a function of *n*).

#### Theorem 2.1

Assume that the process with generator (7) is self-dual with simple factorized self-duality function $D(\xi ,\eta )=\prod d(\xi _x,\eta _x)$ in the form (4) with $d(0,\cdot )\equiv 1$ as in (5). If *d*(1, *n*) is not constant as a function of *n*, then

9 $$\begin{aligned} u(n)= & {} n \nonumber \\ v(n)= & {} v(0)+ (v(1)-v(0))n, \end{aligned}$$

and the first single-site self-duality function is of the form

10 $$\begin{aligned} d(1,n)=a + b n, \end{aligned}$$

for some $a \in {\mathbb {R}}$ and $b \ne 0$.

#### Proof

Using the self-duality relation for $\xi _x = 1$ and no particles elsewhere, together with $u(0)=0$, we obtain the identity

11 $$\begin{aligned}&u(\eta _x)v(\eta _y) (d(1,\eta _x-1)- d(1,\eta _x)) + u(\eta _y)v(\eta _x) (d(1,\eta _x+1)-d(1,\eta _x))\nonumber \\&\quad = p(x,y) u(1)v(0)( d(1,\eta _y)-d(1,\eta _x)) . \end{aligned}$$

Setting $\eta _x=\eta _y=n \ge 1$, this yields anytime $u(n)v(n) \ne 0$

12 $$\begin{aligned} d(1,n+1)+d(1,n-1)-2 d(1,n)=0, \end{aligned}$$

from which we derive $d(1,n)= a+ bn$. Because *d*(1, *n*) is not constant as a function of *n*, we must have $b\not =0$. Inserting $d(1,n)= a+ bn$ in (11) we obtain

13 $$\begin{aligned} u(\eta _x)v(\eta _y)-u(\eta _y)v(\eta _x)= & {} -u(1)v(0)(\eta _y-\eta _x), \end{aligned}$$

from which, by setting $\eta _x=n$ and $\eta _y=0$ we obtain the first in (9), while via $\eta _x=n$ and $\eta _y=1$ we get the second condition. $\square$

#### Remark 2.2

More generally, if we replace (5) with $d(0,n)\ne 0$ in the above statement, we analogously obtain (9) and

14 $$\begin{aligned} d(0,n)= & {} c^n \end{aligned}$$

15 $$\begin{aligned} d(1,n)= & {} (a+bn) \cdot c^n, \end{aligned}$$

for some constants $a, b, c \in {\mathbb {R}}, b, c \ne 0$.

### Interacting Diffusion Systems as Scaling Limits

Conservative interacting diffusion processes arise as scaling limits of the particle systems in Sect. 2.4 (cf. [9]). More in details, by “scaling limit” we refer to the limit process of the particle systems $\{\tfrac{1}{N}\eta ^N(t), t \ge 0 \}_{N \in {\mathbb {N}}}$, where the initial conditions $\tfrac{1}{N}\eta ^N(0)= \tfrac{1}{N}(\lfloor z_x N \rfloor )_{x \in V}$ converge to some $z \in E^V$, with $E = {\mathbb {R}}_+$.

In case of $\text {IRW}$, one obtains a deterministic (hence degenerate diffusion) process $\{z(t), t \ge 0 \}$ whose evolution is described by a first-order differential operator. In case of $\text {SIP}(\alpha )$, the scaling limit is a proper Markov process of interacting diffusions known as Brownian Energy Process ($\text {BEP}(\alpha )$) (cf. [9]). For the $\text {SEP}(\gamma )$, this limit cannot be taken in the sense of Markov processes, but we can extend the SEP generator to a larger class of functions defined on a larger configuration space and take the many-particle limit. The limiting second-order differential operator is then not a Markov generator, but still a second order differential operator. We will explain this more in detail below.

The limiting differential operators in the case of $\text {IRW}$ and $\text {SIP}(\alpha )$ can be described as acting on smooth functions $f : E^V \rightarrow {\mathbb {R}}$ as follows:

16 $$\begin{aligned} {{\mathscr {L}}}f(z)= & {} \sum _{x,y \in V} p(x,y) {{\mathscr {L}}}_{x,y} f(z),\quad z \in E^V, \end{aligned}$$

with single-edge generators ${{\mathscr {L}}}_{x,y}$ given, respectively, by

17 $$\begin{aligned} {{\mathscr {L}}}_{x,y} f(z)= & {} [-(z_x - z_y) (\partial _x-\partial _y) ] f(z)\ ,\quad z \in E^V, \end{aligned}$$

and

18 $$\begin{aligned} {{\mathscr {L}}}_{x,y} f(z)= & {} [-\alpha (z_x - z_y) (\partial _x-\partial _y) + z_x z_y (\partial _x-\partial _y)^2] f(z),\quad z \in E^V. \end{aligned}$$

For the $\text {SEP}(\gamma )$ we proceed as follows. For each $N \in {\mathbb {N}}$, consider the operator $L^N$ working on functions $f : ({\mathbb {N}}/N)^V \rightarrow {\mathbb {R}}$ as

19 $$\begin{aligned} L^N f(\tfrac{1}{N}\eta )= & {} \sum _{x,y\in V} p(x,y) L^N_{x,y}f(\tfrac{1}{N}\eta ),\quad \eta \in {\mathbb {N}}^V, \end{aligned}$$

where

20 $$\begin{aligned} L^N_{x,y} f(\tfrac{1}{N}\eta )= & {} \eta _x(\gamma -\eta _y) (f(\tfrac{1}{N}\eta ^{x,y})-f(\tfrac{1}{N}\eta )) +\eta _y(\gamma -\eta _x)\nonumber \\&(f(\tfrac{1}{N}\eta ^{y,x})-f(\tfrac{1}{N}\eta )),\quad \eta \in {\mathbb {N}}^V. \end{aligned}$$

This operator is not a Markov generator anymore, because the factors $\eta _x(\gamma -\eta _y)$ can become negative. With this operator, we consider the limit

21 $$\begin{aligned} \lim _{N \rightarrow \infty } (L^N f)(\tfrac{1}{N}\eta ^N), \end{aligned}$$

where $\eta ^N= (\lfloor N z_x\rfloor )_{x \in V}$ and $f : E^V \rightarrow {\mathbb {R}}$ is a smooth function. This then gives the differential operator ${{\mathscr {L}}}$ which is the analogue of (16) in the context of $\text {SEP}(\gamma )$. This differential operator ${{\mathscr {L}}}$, with single-edge operators

22 $$\begin{aligned} {{\mathscr {L}}}_{x,y}f(z)= & {} [-\gamma (z_x-z_y)(\partial _x-\partial _y) - z_x z_y(\partial _x-\partial _y)^2] f(z),\quad z \in E^V, \end{aligned}$$

does not generate a Markov process but it is still useful because, as we will see in Sect. 5 below, via generating functions, it is intertwined with the operator (19) for the choice $N=1$.

#### Remark 2.3

The existence and ergodic properties of diffusion processes with generator of type (16) in the context of infinite volume $V={\mathbb {Z}}^d$ has been addressed in [10], Sect. 3, Proposition 3.1, Lemma 3.2, Example 3.4) (this is the proof of the existence of the so-called BMP process in infinite volume, the BEP process is then obtained from a transformation of the BMP process [9], Theorem 6.1]. In the finite-volume case, the process is a multi-type Wright-Fisher diffusion with mutation, of which the existence is well-known.

Naturally, as we can see for the case of $\text {SIP}(\alpha )$ and $\text {BEP}(\alpha )$, when going to the scaling limit, some properties concerning stationary measures and duality pass to the limit. Indeed, $\text {BEP}(\alpha )$ admits a one-parameter family of stationary product measures $\{\otimes \ \nu _\lambda ,\ \lambda > 0 \}$, where $\nu _\lambda \sim \text {Gamma}(\alpha ,\lambda )$, namely

23 $$\begin{aligned} \nu _\lambda (dz)= & {} z^{\alpha -1} e^{-\lambda z} \frac{\lambda ^\alpha }{\Gamma (\alpha )} dz, \end{aligned}$$

and is dual to $\text {SIP}(\alpha )$ with simple factorized duality function $D(\xi ,z)=\prod _{x \in V}d(\xi _x, z_x)$ given by

24 $$\begin{aligned} d(k,z)= & {} z^k \frac{\Gamma (\alpha )}{\Gamma (\alpha +k)},\quad k \in {\mathbb {N}},\quad z \in {\mathbb {R}}_+. \end{aligned}$$

After noting that property (5) holds also in this situation, we show that the first single-site duality functions *d*(1, *x*) between $\text {SIP}(\alpha )$ and $\text {BEP}(\alpha )$ are affine functions of $z \in {\mathbb {R}}_+$, as we found earlier for single-site self-dualities in Theorem 2.1.

#### Proposition 2.1

Assume that $\text {SIP}(\alpha )$ and $\text {BEP}(\alpha )$’s single-edge generators are dual with simple factorized duality function $D(\xi ,z)=\prod d(\xi _x, z_x)$ with $d(0,\cdot )\equiv 1$ as in (4)–(5). Then

25 $$\begin{aligned} d(1,z)= & {} a + b z, \end{aligned}$$

for some $$a, b \in {\mathbb {R}}$$.

#### Proof

The duality relation for $\xi _x = 1$ and no particles elsewhere, by using (5), reads

26 $$\begin{aligned} d(1,z_y) - d(1, z_x)= & {} -\alpha (z_x - z_y) \partial _x d(1,z_x) + z_x z_y \partial _x^2 d(1,z_x). \end{aligned}$$

If we set $z_x = z_y = z$, then $z^2 \frac{d^2}{dz^2} d(1,z)=0$ leads to (25) as unique solution. $\square$

## Relation Between Simple Factorized Duality Functions and Stationary Product Measures

In the examples of duality that we have encountered in the previous section, we have a universal relation between the stationary product measures and the simple factorized duality functions. Given $\{\eta (t),\ t \ge 0 \}$, if there is a dual process $\{\xi (t),\ t \ge 0 \}$ with simple factorized duality functions $D(\xi ,\eta )=\prod d(\xi _x,\eta _x)$ as in (4)–(5) and stationary product measures $\{\mu _\lambda = \otimes \ \nu _\lambda ,\ \lambda > 0\}$, then there is a relation between these measures and these functions, namely there exists a function $\theta (\lambda )$ such that

27 $$\begin{aligned} \int D(\xi ,\eta ) \mu _\lambda (d\eta ) = \prod _{x \in V} \int d(\xi _x,\eta _x) \nu _\lambda (d\eta _x)= & {} \theta (\lambda )^{|\xi |}. \end{aligned}$$

This function $\theta (\lambda )$ is then simply the expectation of the first single-site duality function, i.e.

28 $$\begin{aligned} \theta (\lambda )= & {} \int d(1,\eta _x) \nu _\lambda ( d\eta _x). \end{aligned}$$

In the examples of Sect. 2.4, we have $\theta (\lambda )=\lambda$ for $\text {IRW}$, $\theta (\lambda )=\tfrac{\lambda }{1-\lambda }$ for $\text {SIP}(\alpha )$ and $\theta (\lambda )=\tfrac{\lambda }{1+\lambda }$ for $\text {SEP}(\gamma )$.

In this section we first investigate under which general conditions this relation holds, and further use it in Sects. 3.2.1–3.2.2 as a criterion of characterization of all extremal measures. We refer to Sects. 2.1–2.2 for the general setting in which these results hold.

Later on, we will see that this relation (27) is actually a characterizing property of the simple factorized duality functions, meaning that all duality functions are determined once the first single-site duality function is fixed.

**Main Results of the Section** Theorem 3.1 establishes the equivalence between existence of a stationary product measure and (27) in the finite-volume context, while Theorem 3.2 establishes the same equivalence in the infinite-volume context ($V={\mathbb {Z}}^d$) under the condition [BHT] (bounded harmonic triviality) defined below. As a consequence, in the same infinite-volume context, we obtain Theorems 3.3–3.4 stating that, under [BHT] and existence of simple factorized duality, the only ergodic invariant measures are product measures.

### Finite Case

We start with the simplest situation in which *V* is a finite set.

First, we assume that the total number of particles/the total energy of the dual process is the only conserved quantity. More precisely, we assume the following property, which we refer to as *harmonic triviality* of the dual system:

HT If $H:{{\hat{E}}}^V\rightarrow {\mathbb {R}}$ is harmonic, i.e. such that, for all $t>0$,
  29 $$\begin{aligned} {\hat{{\mathbb {E}}}}_\xi H(\xi (t))= & {} H(\xi ), \end{aligned}$$
  then $H(\xi )$ is only a function of $|\xi |:=\sum _{x\in V} \xi _x$.

Moreover, let us assume the simple factorized form of the duality function $D(\xi , \eta )$ as in (4)–(5). Of the single-site functions *d* we may require the following additional property:

MD The function *d* is *measure determining*, i.e. for two probability measures $\nu _*, \nu _\star$ on *E* such that for all $x\in V$ and $\xi _x\in {\hat{E}}$
  30 $$\begin{aligned} \int _E d(\xi _x, \eta _x)\nu _*(d\eta _x)= & {} \int _E d(\xi _x,\eta _x)\nu _\star (d\eta _x)<\infty , \end{aligned}$$
  it follows that $\nu _*=\nu _\star$.

Then we have the following.

#### Theorem 3.1

Assume that $\{\eta (t),\ t\ge 0\}$ and $\{\xi (t),\ t \ge 0\}$ are dual as in (1) with simple factorized duality function (4) satisfying condition (5). Moreover, assume that [HT] holds and that $\mu$ is a probability measure on $\Omega$. We distinguish two cases:

1. *Interacting particle system case.* If ${\hat{E}}$ is a subset of ${\mathbb {N}}$, then we assume that the self-duality functions $D(\xi , \cdot )$ are $\mu$-integrable for all $\xi \in {\hat{\Omega }}$.
2. *Interacting diffusion case.* If ${\hat{E}}={\mathbb {R}}_+$, then we assume the following integrability condition: for each $\varepsilon > 0$, there exists a $\mu$-integrable function $f_\varepsilon$ such that
  31 $$\begin{aligned} \sup _{\xi \in {\hat{\Omega }},\ |\xi |=\varepsilon } |D(\xi ,\eta )|\le & {} f_\varepsilon (\eta ),\quad \eta \in \Omega . \end{aligned}$$

Then

- $\mu$ is a stationary product measure for the process $\{\eta (t):t\ge 0\}$

implies

(b) For all $\xi \in \Omega$ and for all $x\in V$, we have
  32 $$\begin{aligned} \int D(\xi ,\eta ) \mu (d\eta )= & {} \left( \int D(\delta _x, \eta ) \mu (d\eta )\right) ^{|\xi |}, \end{aligned}$$

where $\delta _x$ denotes the configuration with a single particle at $x \in V$ and no particles elsewhere. Moreover, if condition [MD] holds, the two statements (a) and (b) are equivalent.

#### Proof

First assume that $\mu$ is a stationary product measure. Define $H(\xi )=\int D(\xi ,\eta ) \mu (d\eta )$. By $\mu$-integrability in the interacting particle system case (resp. (31) in the interacting diffusion case), self-duality and invariance of $\mu$ we have

33 $$\begin{aligned} {\mathbb {E}}_\xi H(\xi (t))= & {} \int {\mathbb {E}}_\xi D(\xi (t),\eta ) \mu (d\eta ) = \int {\mathbb {E}}_\eta D(\xi ,\eta (t)) \mu (d\eta )\nonumber \\= & {} \int D(\xi ,\eta ) \mu (d\eta ) =H(\xi ). \end{aligned}$$

Therefore by [HT] we conclude that $H(\xi )=\psi (|\xi |)$. By using $d(0,\cdot )\equiv 1$ and the factorization of the duality functions, we have that $\psi (0)=1$. For the particle case, we obtain that

34 $$\begin{aligned} \int D(\delta _x, \eta ) \mu (d\eta )= & {} \psi (1). \end{aligned}$$

In particular, we obtain that the l.h.s. does not depend on *x*. Next, for $n\ge 2$, put

35 $$\begin{aligned} \int D(n\delta _x, \eta ) \mu (d\eta )= & {} \psi (n), \end{aligned}$$

then we have for $x\not =y \in V$, using the simple factorized duality function and the product form of the measure,

36 $$\begin{aligned} \psi (n)= & {} \int D(n\delta _x, \eta ) \mu (d\eta ) \nonumber \\ {}= & {} \int D(\delta _y,\eta ) D((n-1)\delta _x, \eta ) \mu (d\eta ) = \psi (1) \psi (n-1), \end{aligned}$$

from which it follows that $\psi (n)= \psi (1)^n$. Via an analogous reasoning that uses the factorization of $D(\xi ,\eta )$ and the product form of $\mu$, for the diffusion case we obtain, for all $\varepsilon , \rho \ge 0$,

37 $$\begin{aligned} \psi (\varepsilon +\rho )= & {} \psi (\varepsilon ) \psi (\rho ), \end{aligned}$$

and hence, by measurability of $\psi (\varepsilon )$, we get $\psi (\varepsilon )=\psi (1)^\varepsilon$.

To prove the other implication, put

38 $$\begin{aligned} \int D(\delta _x, \eta ) \mu (d\eta )= & {} \kappa . \end{aligned}$$

We then have by assumption

39 $$\begin{aligned} \int D(\xi ,\eta )\mu (d\eta )= & {} \kappa ^{|\xi |}, \end{aligned}$$

and so it follows that $\mu$ is stationary by self-duality, $\mu$-integrability, the conservation of the number of particles and the measure-determining property. Indeed,

40 $$\begin{aligned} \int {\mathbb {E}}_\eta D(\xi ,\eta (t)) \mu (d\eta )= & {} \int {\mathbb {E}}_\xi D(\xi (t),\eta ) \mu (d\eta ) = {\mathbb {E}}_\xi (\kappa ^{|\xi (t)|})\nonumber \\= & {} \kappa ^{|\xi |}=\int D(\xi ,\eta ) \mu (d\eta ). \end{aligned}$$

From the factorized form of $D(\xi ,\eta )$, (32) implies that for all $x\in V$ and $\xi _x\in {\hat{E}}$

41 $$\begin{aligned} \int d(\xi _x,\eta _x)\mu (d\eta )= & {} \kappa ^{\xi _x}, \end{aligned}$$

and also

42 $$\begin{aligned} \int D(\xi ,\eta )\mu (d\eta ) = \kappa ^{|\xi |} = \prod _{x\in V}\kappa ^{\xi _x} = \prod _{x\in V}\int d(\xi _x,\eta _x)\mu (d\eta )\ , \end{aligned}$$

therefore $\mu$ is a product measure by the fact that *d* is measure determining. $\square$

### Infinite Case

If $V={\mathbb {Z}}^d$, then one needs essentially two extra conditions to state an analogous result in which a general relation between duality functions and corresponding stationary measures can be derived.

In this section we will assume that the dual process is a discrete particle system, i.e. ${\hat{E}}$ is a subset of ${\mathbb {N}}$, in which the number of particles is conserved. In this case we need an additional property ensuring that for the dynamics of a finite number of particles there are no bounded harmonic functions other than those depending on the total number of particles. Therefore, we introduce the condition of existence of a successful coupling for the discrete dual process with a finite number of particles. This is defined below.

#### Definition 3.1

We say that the discrete dual process $\{\xi (t),\ t\ge 0\}$ has the *successful coupling property* when the following holds: if we start with *n* particles then there exists a labeling such that for the corresponding labeled process $\{X_1(t), \ldots , X_n(t),\ t\ge 0\}$ there exists a successful coupling. This means that for every two initial positions $\mathbf{x}=(x_1,\ldots , x_n)$ and $\mathbf{y}=(y_1,\ldots , y_n)$, there exists a coupling with path space measure $\mathbb {P}_\mathbf{x,y}$ such that the coupling time

43 $$\begin{aligned} \tau= & {} \inf \{s>0:\ \mathbf{X}(t)=\mathbf{Y}(t),\ \forall t\ge s \} \end{aligned}$$

is finite $\mathbb {P}_\mathbf{x,y}$ almost surely.

Notice that the successful coupling property is the most common way to prove the following equivalent property (cf. [15]), which is the analogue of [HT], referred here to as *bounded harmonic triviality* of the dual process:

[BHT] If *H* is a bounded harmonic function, then $H(\xi )=\psi (|\xi |)$ for some bounded $\psi : {\hat{E}} \rightarrow {\mathbb {R}}$.

#### Remark 3.1

The condition of the existence of a successful coupling (and the consequent bounded harmonic triviality) is quite natural in the context of interacting particle systems, where we have that a finite number of walkers behave as independent walkers, except when they are close and interact. Therefore, the successful coupling needed is a variation of the Ornstein coupling of independent walkers, see e.g. [3, 5, 14].

Furthermore, we need a form of uniform $\mu$-integrability of the duality functions which we introduce below and call *uniform domination property* of *D* w.r.t. $\mu$ (note the analogy with condition (31)):

[UD] Given $\mu$ a probability measure on $\Omega$, the duality functions $\{D(\xi ,\cdot ),\ |\xi |=n\}$ are uniformly $\mu$-integrable, i.e. for all $n \in {\mathbb {N}}$ there exists a function $f_n$ such that $f_n$ is $\mu$-integrable and such that for all $\eta \in \Omega$
  44 $$\begin{aligned} \sup _{\xi \in {\hat{\Omega }},\ |\xi |=n} |D(\xi , \eta )|\le f_n(\eta ). \end{aligned}$$

Under these conditions, the following result holds, whose proof resembles that of Theorem 3.1.

#### Theorem 3.2

Assume as in (1) that $\{\eta (t),\ t\ge 0\}$ is dual to the discrete process $\{\xi (t),\ t \ge 0 \}$ with simple factorized duality function as in (4)–(5). Moreover, assume [BHT] in place of [HT] for the dual process and that $\mu$ is a probability measure on $\Omega$ such that [UD] holds. Then the same conclusions as in Theorem 3.1 follow, where (32) holds for all finite configurations $\xi \in {\hat{\Omega }}$.

#### Translation Invariant Case

In this section, we show that under the assumption of simple factorized duality functions, minimal ergodicity assumptions on a stationary probability measure $\mu$ on $\Omega$ are needed to ensure (32) and, as a consequence, that $\mu$ is product measure.

Here we restrict to the case $V={\mathbb {Z}}^d$ because we will use spatial ergodicity.

##### Theorem 3.3

In the setting of Theorem 3.2 with $D(\xi ,\eta )$ simple factorized duality function and $\mu$ probability measure on $\Omega$, if $\mu$ is a translation-invariant and ergodic (under translations) stationary measure for $\{\eta (t),\ t \ge 0 \}$, then we have (32) for all finite configurations $\xi$; as a consequence, $\mu$ is a product measure.

##### Proof

To start, let us consider a configuration $\xi =\sum _{i=1}^n \delta _{x_i}$. By bounded harmonic triviality [BHT], combined with the bound (44) for all such configurations, $\int D(\xi , \eta ) \mu (d\eta )$ is only depending on *n* and, therefore, we can replace $\xi$ by $\sum _{i=1}^{n-1} \delta _{x_i} +\delta _y$, where *y* is arbitrary in ${\mathbb {Z}}^d$. Let us call $B_N=[-N,N]^d\cap {\mathbb {Z}}^d$. Fix $N_0$ such that $B_{N_0}$ contains all the points $x_1,\ldots x_{n-1}$. For *y* outside $B_{N_0}$, by the factorization property, $D(\sum _{i=1}^{n-1} \delta _{x_i}+\delta _y, \eta )=D(\sum _{i=1}^{n-1} \delta _{x_i}, \eta ) D(\delta _y, \eta )$. By the Birkhoff ergodic theorem, we have that

45 $$\begin{aligned} \frac{1}{(2N+1)^d}\sum _{y\in B_N} D(\delta _y, \eta )\rightarrow & {} \int D(\delta _0, \eta )\mu (d\eta ) \end{aligned}$$

$\mu$-a.s. as $N\rightarrow \infty$. Using this, together with (44), we have

46 $$\begin{aligned} \int D(\xi , \eta ) \mu (d\eta )= & {} \lim _{N\rightarrow \infty }\frac{1}{(2N+1)^d}\sum _{y\in B_N\setminus B_{N_0}} \int D\left( \sum _{i=1}^{n-1} \delta _{x_i}, \eta \right) D(\delta _y, \eta )\mu (d\eta ) \nonumber \\= & {} \int D\left( \sum _{i=1}^{n-1} \delta _{x_i}, \eta \right) \mu (d\eta ) \int D(\delta _0, \eta )\mu (d\eta ). \end{aligned}$$

Iterating this argument gives (32). $\square$

##### Remark 3.2

As follows clearly from the proof, the condition of factorization of the duality function can be replaced by the weaker condition of

47 $$\begin{aligned} \lim _{|y|\rightarrow \infty } (D(\delta _{x_1}+\cdots + \delta _{x_n} + \delta _y, \eta )- D(\delta _{x_1}+\cdots + \delta _{x_n},\eta ) D( \delta _y, \eta ))= & {} 0, \end{aligned}$$

for all $\eta , x_1,\ldots ,x_n$. We note that this approximate factorization of the duality function leads to (32), though $\mu$ is not necessarily a product measure.

#### Non-translation Invariant Case

We continue here with $V={\mathbb {Z}}^d$ but drop the assumption of translation invariance. Indeed, equality (32) is also valid in contexts where one cannot rely on translation invariance. Examples include spatially inhomogeneous $\text {SIP}(\varvec{\alpha })$ and $\text {SEP}(\varvec{\gamma })$, where the parameters $\varvec{\alpha }= (\alpha _x)_{x \in V}$ and $\varvec{\gamma }= (\gamma _x)_{x \in V}$ in Sect. 2.3 may depend on the site accordingly (cf. e.g. [17]). Also in this inhomogeneous setting the self-duality functions factorize over sites and the stationary measures are in product form, with site-dependent single-site duality functions, resp. site-dependent marginals. We will show that the relation (32) between the self-duality functions and any ergodic stationary measure still holds, and as a consequence this ergodic stationary measures is in fact a product measure. The idea is that the averaging over space w.r.t. $\mu$, used in the proof of Theorem 3.3 above, can be replaced by a time average.

If we start with a single dual particle, the dual process is a continuous-time random walk on *V*, for which we denote by *p*(*t*; *x*, *y*) the transition probability to go from *x* to *y* in time $t>0$. A basic assumption will then be

48 $$\begin{aligned} \lim _{t\rightarrow \infty } p(t; x,y)=0 \end{aligned}$$

for all $$x, y \in V$$.

##### Theorem 3.4

In the setting of Theorem 3.2 with $D(\xi ,\eta )$ duality function and $\mu$ probability measure on $\Omega$, if $\mu$ is an ergodic stationary measure for the process $\{\eta (t),\ t \ge 0 \}$ and (48) holds for the dual particle, then we have (32) for all finite configurations $\xi$; as a consequence, $\mu$ is a product measure.

##### Proof

The idea is to replace the spatial average in the proof of Theorem 3.3 by a Cesaro average over time, which we can deal by combining assumption (48) with the assumed temporal ergodicity.

Fix $$x_1,\ldots ,x_n\in V, y\in V$$. Define

49 $$\begin{aligned} H_{n+1}(x_1,\ldots ,x_n,y)= & {} \int D(\delta _{x_1}+ \ldots +\delta _{x_n}+ \delta _y,\eta ) \mu (d\eta ) \end{aligned}$$

50 $$\begin{aligned} H_1(y)= & {} \int D(\delta _y,\eta ) \mu (d\eta ) \end{aligned}$$

51 $$\begin{aligned} H_{n}(x_1,\ldots ,x_n)= & {} \int D(\delta _{x_1}+ \ldots +\delta _{x_n},\eta ) \mu (d\eta ). \end{aligned}$$

It is sufficient to obtain that $H_{n+1}(x_1,\ldots ,x_n,y)= H_1(y)H_{n}(x_1,\ldots ,x_n)$. We already know by the bounded harmonic triviality that $H_{n}$ only depends on *n* and not on the given locations $x_1,\ldots ,x_n$. Therefore, we have

52 $$\begin{aligned} H_{n+1}(x_1,\ldots ,x_n,y)= & {} \sum _{z} p(t; y,z) H_{n+1}(x_1,\ldots ,x_n,z). \end{aligned}$$

By assumption (48), this implies

53 $$\begin{aligned} H_{n+1}(x_1,\ldots ,x_n,y)= & {} \lim _{T\rightarrow \infty }\frac{1}{T} \int _0^T dt \sum _{z\not \in \{x_1,\ldots ,x_n\}} p(t; y,z) H_{n+1}(x_1,\ldots ,x_n,z) \nonumber \\= & {} \lim _{T\rightarrow \infty }\frac{1}{T} \int _0^T dt \sum _{z\not \in \{x_1,\ldots ,x_n\}} p(t; y,z) \int D(\delta _{x_1}+ \ldots +\delta _{x_n},\eta )D( \delta _y,\eta ) \mu (d\eta ) \nonumber \\= & {} \lim _{T\rightarrow \infty }\frac{1}{T} \int _0^T dt \sum _{z} p(t; y,z) \int D(\delta _{x_1}+ \ldots +\delta _{x_n},\eta )D( \delta _y,\eta ) \mu (d\eta ) \nonumber \\= & {} \lim _{T\rightarrow \infty }\frac{1}{T}\int _0^T dt \int D(\delta _{x_1}+ \ldots +\delta _{x_n},\eta ){\mathbb {E}}_\eta D( \delta _y,\eta (t)) \mu (d\eta ) \nonumber \\= & {} H_1(y)H_{n}(x_1,\ldots ,x_n), \end{aligned}$$

where in the last step we used the assumed temporal ergodicity of $\mu$ and Birkhoff ergodic theorem. $\square$

## From Stationary Product Measures to Duality Functions

As we have just illustrated in Sects. 3.2.1–3.2.2, relation (27) turns out to be useful in deriving information about the product structure of stationary ergodic measures from the knowledge of simple factorized duality functions. On the other side, granted some information on the stationary product measures, which follows usually from a simple detailed balance computation, up to which extent does relation (27) say something about the possible simple factorized duality functions?

In the context of conditions (4)–(5) and in presence of a one-parameter family of stationary product measures $\{\mu _\lambda = \otimes \ \nu _\lambda ,\ \lambda > 0 \}$, relation (27) for $\xi = k \delta _x \in {\hat{\Omega }}$ for some $k \in {\mathbb {N}}$ reads

54 $$\begin{aligned} \int _E d(k,\eta _x) \nu _\lambda (d\eta _x) = \left( \int _E d(1,\eta _x) \nu _\lambda (d\eta _x) \right) ^k= & {} \theta (\lambda )^k. \end{aligned}$$

As a consequence, knowing the first single-site duality function $d(1,\cdot )$ and the explicit expression of the marginal $\nu _\lambda$ is enough to recover the l.h.s. in (54). However, rather than obtaining $d(k,\cdot )$, at this stage the l.h.s. has still the form of an “integral transform”-type of expression for $d(k,\cdot )$.

In the next two subsections, we show how to recover $d(k,\eta _x)$ from (54) and the knowledge of $\theta (\lambda )$. This then leads to the characterization of all possible simple factorized (self-)duality functions.

**Main Results of the Section** Relation (27), together with the knowledge of the first single-site self-duality function, determines all candidate simple factorized (self)-duality functions. This is shown in Section  4.1 for particle systems (self-duality for Exclusion, Inclusion and Independent Random Walkers) and in Sect. 4.2 for diffusion processes (duality between Inclusion and Brownian Energy processes).

### Particle Systems and Orthogonal Polynomial Self-duality Functions

Going back to the interacting particle systems introduced in Sect. 2.3 with infinitesimal generator (6) and product stationary measures with marginals (8), the integral relation (54) rewrites, for each $k \in {\mathbb {N}}$ and $\lambda > 0$ for which $Z_\lambda < \infty$, as

55 $$\begin{aligned} \sum _{n \in {\mathbb {N}}} d(k,n) \nu _\lambda (n) = \sum _{n \in {\mathbb {N}}} d(k,n) \varphi (n) \frac{\lambda ^n}{n!} \frac{1}{Z_\lambda }= & {} \theta (\lambda )^k, \end{aligned}$$

where $\varphi (n)= n! \prod _{m=1}^n \tfrac{v(m-1)}{u(m)}$. Now, if we interpret

56 $$\begin{aligned} \sum _{n \in {\mathbb {N}}} d(k,n) \varphi (n) \frac{\lambda ^n}{n!} \end{aligned}$$

as the *Taylor series expansion* around $\lambda =0$ of the function $\theta (\lambda )^k Z_\lambda$, we can re-obtain the explicit formula of $d(k,n) \varphi (n)$ as its *n*th order derivative evaluated at $\lambda =0$, namely

57 $$\begin{aligned} d(k,n) \varphi (n)= & {} \left( \left[ \frac{d^n}{d\lambda ^n}\right] _{\lambda =0}\theta (\lambda )^k Z_\lambda \right) , \end{aligned}$$

and hence, anytime $\varphi (n) > 0$,

58 $$\begin{aligned} d(k,n)= & {} \frac{1}{\varphi (n)}\left( \left[ \frac{d^n}{d\lambda ^n}\right] _{\lambda =0}\theta (\lambda )^k Z_\lambda \right) . \end{aligned}$$

Together with the full characterization obtained in Theorem  2.1 of the first single-site self-duality functions $d(1,\cdot )$ - and $\theta (\lambda )$ in turn - we obtain via this procedure a full characterization of all single-site self-duality functions. Beside recovering the “classical" dualities illustrated in Sect. 2.3, we find the single-site self-duality functions in terms of orthogonal polynomials $\{p_k(n),\ k \in {\mathbb {N}}\}$ of a discrete variable (cf. e.g. [16]) recently discovered via a different approach in [8]. We add the observation that all these new single-site self-duality functions can be obtained from the classical ones via a Gram–Schmidt orthogonalization procedure w.r.t. the correct probability measures on ${\mathbb {N}}$, namely the marginals of the associated stationary product measures (cf. [8]).

We divide the discussion in three cases, one suitable for processes of $\text {IRW}$-type, the other for $\text {SIP}$ and $\text {SEP}$ and the last one for the remaining particle systems.

#### Independent Random Walkers

We recall that the $\text {IRW}$-case corresponds to the choice of values in (9) satisfying the relation $v(1)=v(0)$. If we compute $\theta (\lambda )$ for the general first single-site self-duality function $d(1,n)=a+b n$ obtained in (10), we get

59 $$\begin{aligned} \theta (\lambda )= & {} \sum _n (a+b n) \frac{\lambda ^n}{n!}\frac{1}{Z_\lambda }= a + b\lambda . \end{aligned}$$

and, in turn via relation (58), we recover all functions $d(k,\cdot )$, for $k > 1$:

60 $$\begin{aligned} d(k,n)= & {} \left( \left[ \frac{d^n}{d \lambda ^n} \right] _{\lambda =0} \left( a + b\lambda \right) ^k e^\lambda \right) \nonumber \\= & {} \sum _{r=0}^n \left( {\begin{array}{c}n\\ r\end{array}}\right) k (k-1) \cdots (k-r+1) b^r a^{k-r}. \end{aligned}$$

In case $a=0, d(k,n) = 0$ for $n < k$, while for $n \ge k$, in the summation all terms but the one corresponding to $r=k$ vanish, thus $d(k,n)= \frac{n!}{(n-k)!} b^k$. In case $a \ne 0$,61In conclusion, for the choice $a \cdot b < 0$,

62 $$\begin{aligned} d(k,n) = a^k\ C_k(n; -\tfrac{a}{b}), \end{aligned}$$

where $\{C_k(n; \mu ),\ k \in {\mathbb {N}}\}$ are the Poisson–Charlier polynomials - orthogonal polynomials w.r.t. the Poisson distribution of parameter $\mu > 0$ (cf. [16]).

#### Inclusion and Exclusion Processes

For $\text {SIP}$ and $\text {SEP}$ we are in the case $v(1) \ne v(0)$, and hence we abbreviate

63 $$\begin{aligned} \sigma = v(1)-v(0),\quad \beta = v(0), \end{aligned}$$

where for $\text {SIP}(\alpha )$ we choose $\sigma =1$ and $\beta =\alpha$, while for $\text {SEP}(\gamma )$ we set $\sigma =-1$ and $\beta =\gamma$. If we compute $\theta (\lambda )$ for $d(1,n)=a+bn$ in (10), we have

64 $$\begin{aligned} \theta (\lambda )= & {} a + b \beta \lambda \left( 1-\sigma \lambda \right) ^{-1} = \frac{a+\left( b \beta - a \sigma \right) \lambda }{1-\sigma \lambda }. \end{aligned}$$

By applying formula (58), we obtain all functions *d*(*k*, *n*) for $k > 1$ as follows:

65 $$\begin{aligned} d(k,n)= & {} \nonumber \frac{1}{\varphi (n)}\left( \left[ \frac{d^n}{d\lambda ^n}\right] _{\lambda =0}\left( a+\left( b\beta - a \sigma \right) \lambda \right) ^k \left( 1-\sigma \lambda \right) ^{-k-\sigma \beta } \right) \ \nonumber \\= & {} \frac{\Gamma \left( \sigma \beta \right) }{\Gamma \left( \sigma \beta +n \right) } \sum _{r=0}^n \left( {\begin{array}{c}n\\ r\end{array}}\right) \left( {\begin{array}{c}k\\ r\end{array}}\right) r! a^{k-r} \left( b \sigma \beta - a \right) ^{r} \frac{\Gamma \left( \sigma \beta + n + k -r \right) }{\Gamma \left( \sigma \beta + k \right) }. \end{aligned}$$

In case $a=0$, clearly $d(k,n)=0$ for $k < n$, while for $n \ge k$ only the term for $r=k$ is nonzero in the summation:

66 $$\begin{aligned} d(k,n)= & {} \frac{n!}{(n-k)!} \frac{\Gamma \left( \sigma \beta \right) }{\Gamma \left( \sigma \beta + k\right) } \left( b \sigma \beta \right) ^k. \end{aligned}$$

In case $a \ne 0$, by using the known relation (cf. [16], p. 51),67If $\sigma =1, \beta = \alpha > 0$ and if $a \cdot b < 0$, we recognize in (67) the Meixner polynomials as defined in [16], i.e.

68 $$\begin{aligned} d(k,n) = a^k\ \frac{\Gamma \left( \alpha \right) }{\Gamma \left( \alpha + k \right) }\ M_k(n; \mu , \alpha ), \end{aligned}$$

where in our case $\mu = \frac{a}{a-b \alpha }$ and $\{M_k(n; \mu , \alpha ),\ k \in {\mathbb {N}}\}$ are the Meixner polynomials - orthogonal polynomials w.r.t. the discrete Gamma distribution of scale parameter $\mu$ and shape parameter $\alpha$.

Furthermore, if $\sigma =-1, \beta = \gamma$ with $\gamma \in {\mathbb {N}}$ and given the additional requirements $a \cdot b < 0$ and $-\frac{a}{b} \le \gamma$, from the expression in (67) we have a representation of the single-site duality functions in terms of the Kravchuk polynomials as defined in [16], i.e.

69 $$\begin{aligned} d(k,n)= & {} a^k\ K_k(n; p, \gamma )\ \left( -\frac{1}{p} \right) ^k \frac{1}{\left( {\begin{array}{c}\gamma \\ k\end{array}}\right) }, \end{aligned}$$

where $p= -\frac{a}{b \gamma }$ in our case and $\{K_k(n; p, \gamma ),\ k \in {\mathbb {N}}\}$ the Kravchuk polynomials - orthogonal polynomials w.r.t. the Binomial distribution of parameters $\gamma$ and *p*.

As a conclusion of this procedure, we note that all factorized self-duality functions for independent random walkers, inclusion and exclusion processes satisfying (5) are either in the “classical” form of Sect. 2.3 (case $a=0$) or consist of products of rescaled versions of orthogonal polynomials (case $a \ne 0$). Other simple factorized self-duality functions for the systems $\text {IRW}, \text {SIP}$ and $\text {SEP}$ do not exist.

##### Remark 4.1

It is interesting to note that, apart from the leading factor $a^k$, the remaining *polynomials* in the expressions of *d*(*k*, *n*) for $a \ne 0$ are “*self-dual*" in the sense of the orthogonal polynomials literature, i.e. $p_n(k)=p_k(n)$ in our context (cf. Definition 3.1, [13]). Henceforth, if *d*(*k*, *n*) is interpreted as a countable matrix, the value $a \in {\mathbb {R}}$ is the only responsible for the asymmetry of *d*(*k*, *n*): upper-triangular for $a=0$ while symmetric for $a=1$.

#### Trivial Factorized Self-duality

To conclude, for the sake of completeness, we can implement the same machinery to cover all factorized self-dualities with property (5) for all discrete processes of type (6).

Indeed, from the proof of Theorem 2.1, if the process is neither of the types $\text {IRW}, \text {SIP}$ and $\text {SEP}$, then the only possible choice is $d(1,n)=a$ for some $a \in {\mathbb {R}}$, i.e. it is not depending on *n*. From this we get $\theta (\lambda )= a$, and $d(k,n)=a^k$ from formula (58). Hence, the self-duality functions must be of the form

70 $$\begin{aligned} D(\xi ,\eta )= & {} \prod _{x \in V}d(\xi _x,\eta _x) = a^{|\xi |}, \end{aligned}$$

i.e. depending only on the total number of dual particles (and not on the configuration $\eta$). Hence, the duality relation in that case reduces to the trivial relation, for all $t \ge 0$ and $\xi \in {\hat{\Omega }}$,

71 $$\begin{aligned} {\mathbb {E}}_\xi a^{|\xi _t|}= & {} a^{|\xi |},\quad a \in {\mathbb {R}}, \end{aligned}$$

which is just conservation of the number of particles in the dual process. No other self-duality relation with simple factorized self-duality functions can exist.

### Interacting Diffusions and Orthogonal Polynomial Duality Functions

As shown in Theorem 3.1, relation (54) still holds whenever the discrete right-variables $n \in {\mathbb {N}}$ are replaced by continuous variables $z \in {\mathbb {R}}_+$ and sums by integrals. With this observation in mind, we provide a second general method to *characterize all simple factorized duality functions* between the continuous process $\text {BEP}(\alpha )$ and its discrete dual $\text {SIP}(\alpha )$.

More precisely, if *d*(*k*, *z*) is a single-site duality function with property (5) between $\text {BEP}(\alpha )$ and $\text {SIP}(\alpha )$, and $\nu _\lambda$ is the stationary product measure marginal for $\text {BEP}(\alpha )$ as in (23), then, from the analogue of relation (54) for $k=1$, namely

72 $$\begin{aligned} \int _{{\mathbb {R}}_+} d(1,z) z^{\alpha -1} e^{-\lambda z}\frac{\lambda ^\alpha }{\Gamma (\alpha )} dz= & {} \theta (\lambda ), \end{aligned}$$

we necessarily have by Theorem 3.1 that

73 $$\begin{aligned} \int d(k,z) \frac{z^{\alpha -1}}{\Gamma (\alpha )} e^{-\lambda z} dz= & {} \theta (\lambda )^k \lambda ^{-\alpha }\ . \end{aligned}$$

As a consequence, the function $d(k,z) \frac{z^{\alpha -1}}{\Gamma (\alpha )}$ is the inverse Laplace transform of $\theta (\lambda )^k \lambda ^{-\alpha }$. Given the first single-site duality function *d*(1, *z*) in (25), from (72) we obtain

74 $$\begin{aligned} \theta (\lambda )= & {} \int (a+bz) z^{\alpha -1} e^{-\lambda z} \frac{\lambda ^\alpha }{\Gamma (\alpha )} dz = \left( a \lambda + b \alpha \right) \lambda ^{-1}. \end{aligned}$$

As a consequence, the r.h.s. in (73) becomes

75 $$\begin{aligned} \theta (\lambda )^k \lambda ^{-\alpha }= & {} \left( a \lambda + b \alpha \right) ^k \lambda ^{ - \alpha -k}, \end{aligned}$$

and there exist explicit expressions for the inverse Laplace transform of this function. We split the computation in two cases. In case $a=0$, since the inverse Laplace transform of $\lambda ^{-\alpha -k}$ is $\frac{z^{\alpha +k-1}}{\Gamma (\alpha +k)}$, we immediately obtain

76 $$\begin{aligned} d(k,z)= & {} (b \alpha )^k \frac{z^k \Gamma (\alpha )}{\Gamma (\alpha +k)}, \end{aligned}$$

i.e. the “classical” single-site duality function as in Sect. 2.5, up to set $b=\frac{1}{\alpha }$. In case $a \ne 0$, the inverse Laplace transform of (75) is more elaborated:77As the above expression must equal $d(k,z) \frac{z^{\alpha -1}}{\Gamma (\alpha )}$, it follows that78As a final consideration, we note that for the choice $a \cdot b < 0$,

79 $$\begin{aligned} d(k,z)= & {} a^k\ \frac{k! \Gamma (\alpha )}{\Gamma (\alpha +k)}\ L_k(z; \alpha -1, \mu ), \end{aligned}$$

where $\mu = -\frac{b \alpha }{a}$ here and $\{L_k(z; \alpha -1, \mu ),\ k \in {\mathbb {N}}\}$ are the generalized Laguerre polynomials–orthogonal polynomials w.r.t. to the Gamma distribution of scale parameter $\frac{1}{\mu }$ and shape parameter $\alpha$ as defined in [16].

## Intertwining and Generating Functions

In this section, we introduce the generating function method (cf. [9]), which allows to go from a self-duality of a discrete process towards duality between a discrete and continuous process, and further towards a self-duality of a continuous process, and back. This then allows e.g. to simplify the proof of a discrete self-duality by lifting it to a continuous self-duality, which is usually easier to verify. The key of this method is an intertwining between discrete multiplication and derivation operators and their continuous analogues, via an appropriate generating function.

To reduce issues of well-definition of operators and their domains, in this section we restrict our discussion to the case of finite vertex set *V*.

We start with the introductory example of Independent Random Walkers in Sect. 5.1, showing its generator intertwines with a first order differential operator, and from that recover in an easy way the self-duality of independent random walkers with the “classical” self-duality functions.

**Main Results of the Section** After an introduction to intertwining in Sect. 5.2 and its relation with duality in Theorem 5.1, we find in Propositions 5.1–5.2 (product) intertwiners between the discrete particle system generators and their diffusion counterparts. As a corollary of Propositions 5.1–5.2, in Sect. 5.4 we prove that all the candidate simple factorized self-duality functions produced in Sect. 4.1 (from the stationary product measures via (27)) are actual self-duality functions. Seemingly, we also produce several (self-)duality functions for the diffusion counterparts of the particle systems.

### Introductory Example

To make the method clear, let us start with a simple example of independent random walkers on a single edge. The generator is

80 $$\begin{aligned} L f(n_1,n_2)= & {} n_1 (f(n_1-1, n_2+1)- f(n_1,n_2)) \nonumber \\&+\, n_2 (f(n_1+1, n_2-1)- f(n_1,n_2)), \end{aligned}$$

with $n_1, n_2 \in {\mathbb {N}}$. Define now the (exponential) generating function

81 $$\begin{aligned} {{\mathcal {G}}}f(z_1,z_2)= & {} \sum _{n_1,n_2=0}^\infty f(n_1,n_2) \frac{z_1^{n_1}}{n_1!} \frac{z_2^{n_2}}{n_2!},\quad z_1, z_2 \in {\mathbb {R}}_+. \end{aligned}$$

Then it is easy to see that

82 $$\begin{aligned} {{\mathscr {L}}}{{\mathcal {G}}}= & {} {{\mathcal {G}}}L, \end{aligned}$$

where

83 $$\begin{aligned} {{\mathscr {L}}}= & {} -(z_1-z_2)(\partial _{z_1}-\partial _{z_2}). \end{aligned}$$

Now assume that we have a self-duality function for the particle system, i.e.

84 $$\begin{aligned} L_D= & {} L_\mathrm{right}D, \end{aligned}$$

then we have

85 $$\begin{aligned} L_{{\mathcal {D}}}= & {} {{\mathscr {L}}}_\mathrm{right}{{\mathcal {D}}}, \end{aligned}$$

where

86 $$\begin{aligned} {{\mathcal {D}}}(k_1, k_2; z_1, z_2) = {{\mathcal {G}}}_{\mathrm{right}} D(k_1, k_2; z_1, z_2) = \sum _{n_1, n_2=0}^\infty D(k_1,k_2; n_1,n_2) \frac{z_1^{n_1}}{n_1!} \frac{z_2^{n_2}}{n_2!}. \end{aligned}$$

In words, a self-duality function of *L* is “lifted” to a duality function between the independent random walk generator *L* and its continuous counterpart ${{\mathscr {L}}}$ by applying the generating function to the *n*-variables. Conversely, given a duality function between the independent random walk generator *L* and its continuous counterpart ${{\mathscr {L}}}$, its Taylor coefficients provide a self-duality function of *L*.

We can then also take the generating function w.r.t. the *k*-variables in the function ${{\mathcal {D}}}$ to produce a self-duality function for ${{\mathscr {L}}}$, i.e. defining

87 $$\begin{aligned} {\mathscr {D}}(v_1, v_2; z_1, z_2) = {{\mathcal {G}}}_{{\mathcal {D}}}(v_1, v_2; z_1, z_2) = \sum _{k_1, k_2=0}^\infty {{\mathcal {D}}}(k_1, k_2; z_1, z_2) \frac{v_1^{k_1}}{k_1!} \frac{v_2^{k_2}}{ k_2!}, \end{aligned}$$

we have

88 $$\begin{aligned} {{\mathscr {L}}}_{\mathscr {D}}= & {} {{\mathscr {L}}}_\mathrm{right}{\mathscr {D}}. \end{aligned}$$

For the classical self-duality function $D(k_1,k_2; n_1,n_2)= \frac{n_1!}{(n_1-k_1)!}\frac{n_2!}{(n_2-k_2)!}{\mathbb {1}}\{k_1\le n_1\}{\mathbb {1}}\{k_2\le n_2\}$, we find that

89 $$\begin{aligned} {\mathscr {D}}(v_1, v_2; z_1, z_2) = e^{z_1+z_2} e^{v_1 z_1+ v_2 z_2}. \end{aligned}$$

Beside the factor $e^{z_1+z_2}$ which depends only on the conserved quantity $z_1+z_2$, to check the self-duality relation for ${{\mathscr {L}}}$ w.r.t. the function $e^{v_1 z_1+ v_2 z_2}$ is rather straightforward, the computation involving only derivatives of exponentials. By looking at the Taylor coefficients w.r.t. both *v* and *z*-variables of this self-duality relation, we obtain the self-duality relation for *L* w.r.t. *D* where we started from.

In conclusion, all these duality relations turn out to be equivalent, and the proof of self-duality for particle systems requiring rather intricate combinatorial arguments (cf. e.g. [3]) is superfluous once the more direct self-duality for diffusion systems is checked.

### Intertwining and Duality

The notion of *intertwining* between stochastic processes was originally introduced by Yor in [20] in the context of Markov chains and later pursued in [4] and [6] as an abstract framework, in discrete-time and continuous-time respectively, for the problem of Markov functionals, i.e. finding sufficient and necessary conditions under which a random function of a Markov chain is again Markovian.

For later purposes, we adopt a rather general definition of intertwining, in which $\{\eta (t),\ t \ge 0 \}$ and $\{\zeta (t),\ t \ge 0 \}$ are continuous-time stochastic processes on the Polish spaces $\Omega$ and $\Omega '$, respectively, whose expectations read ${\mathbb {E}}, {\mathbb {E}}'$ resp., and ${{\mathcal {M}}}( \Omega )$ denotes the space of signed measures on $\Omega$. We say that $\{\zeta (t),\ t \ge 0 \}$ is *intertwined on top* of $\{\eta (t),\ t \ge 0 \}$ if there exists a mapping $\varLambda : \Omega ' \rightarrow {{\mathcal {M}}}(\Omega )$ such that, for all $t \ge 0, \zeta \in \Omega '$ and $f : \Omega \rightarrow {\mathbb {R}}$,

90 $$\begin{aligned} {\mathbb {E}}'_\zeta \int _{\Omega '} f(\eta )\ \varLambda (\zeta (t))(d\eta )= & {} \int _{\Omega } {\mathbb {E}}_{\eta } f(\eta (t))\ \varLambda (\zeta )(d\eta ). \end{aligned}$$

Working at the abstract level of *semigroups*, we say that $\{{{\mathscr {S}}}(t),\ t \ge 0 \}$ on a space of functions $f:\Omega '\rightarrow {\mathbb {R}}$ denoted by ${{\mathcal {F}}}(\Omega ')$, is *intertwined on top* of $\{S(t),\ t \ge 0 \}$, a semigroup on a space of functions $f:\Omega \rightarrow {\mathbb {R}}$ denoted by ${{\mathcal {F}}}(\Omega )$, with *intertwiner* $\varLambda$ if $\varLambda$ is a linear operator from ${{\mathcal {F}}}(\Omega )$ into ${{\mathcal {F}}}(\Omega ')$ and if, for all $t \ge 0$ and $f : \Omega \rightarrow {\mathbb {R}}$,

91 $$\begin{aligned} {{\mathscr {S}}}(t) \varLambda f= & {} \varLambda S(t) f. \end{aligned}$$

Similarly, *operators* ${{\mathscr {L}}}$ with domain ${{\mathcal {D}}}({{\mathscr {L}}})$ and *L* with domain ${{\mathcal {D}}}(L)$ are *intertwined* with intertwiner $\varLambda$ if, for all $f \in {{\mathcal {D}}}(L), \varLambda f\in {{\mathcal {D}}}({{\mathscr {L}}})$ and

92 $$\begin{aligned} {{\mathscr {L}}}\varLambda f= & {} \varLambda L f. \end{aligned}$$

Notice that with a slight abuse of notation we used the same symbol $\varLambda$ for an abstract intertwining operator as for the intertwining mapping. In other words, in case the intertwining mapping as in (90) is given by ${\tilde{\varLambda }}$, then the corresponding operator is

93 $$\begin{aligned} \varLambda f(\zeta )= & {} \int f(\eta )\ {{\tilde{\varLambda }}(\zeta )}(d\eta ). \end{aligned}$$

An intertwining mapping $\varLambda$ has a probabilistic interpretation if it takes values in the subset of probability measures on $\Omega$. Indeed, in (90) the process $\{\zeta (t),\ t \ge 0 \}$ may be viewed as an added structure on top of $\{\eta (t),\ t \ge 0 \}$ or, alternatively, the process $\{\eta (t),\ t \ge 0 \}$ as a random functional of $\{\zeta (t),\ t \ge 0\}$, in which $\varLambda$ provides this link.

#### Remark 5.1

The connection with duality introduced in Sect. 2.1 becomes transparent when $\Omega , {\hat{\Omega }}$ and $\Omega '$ are finite sets and the operators and functions *L*, ${\hat{L}} {{\mathscr {L}}}, D$ and $\varLambda$ in (3) and (92) are represented in terms of matrices. There, relations (3) and (92), once rewritten in matrix notation as

94 $$\begin{aligned} {\hat{L}} D= & {} D L^\dagger , \end{aligned}$$

where $L^\dagger$ denotes the transpose of *L*, and

95 $$\begin{aligned} {{\mathscr {L}}}\varLambda= & {} \varLambda L, \end{aligned}$$

differ essentially only in the terms $L^\dagger$ versus *L* in the r.h.s. of both identities. The presence or absence of transposition can be interpreted as a *forward*-versus-*backward* evolution against a *forward*-versus-*forward* evolution. More precisely, if $L, {\hat{L}}$ and ${{\mathscr {L}}}$ are generators of Markov processes $\{\eta (t), t \ge 0\}, \{\xi (t),\ t \ge 0 \}$ and $\{\zeta (t),\ t \ge 0 \}$, respectively, then (94) and (95) relate the evolution of $\{\eta (t),\ t \ge 0\}$ to that of $\{\xi (t),\ t \ge 0 \}$, resp. $\{\zeta (t),\ t \ge 0 \}$; however, while in (95) the processes run both along the same direction in time, in (94) the processes run along opposite time directions.

Intertwiners as $\varLambda$ in (95) may be also interpreted as natural generalizations of *symmetries of generators*, indeed (95) with ${{\mathscr {L}}}= L$ just means that $\Lambda$ commutes with *L*, which is the definition of a symmetry of *L*. As outlined in [9, Theorem 2.6], the knowledge of symmetries of a generator and dualities of this generator leads to the construction of new dualities. The following theorem presents the analogue procedure in presence of intertwiners: a duality and an intertwining lead to a new duality.

#### Theorem 5.1

Let $L, {\hat{L}}$ and ${{\mathscr {L}}}$ be operators on real-valued functions on $\Omega , {\hat{\Omega }}$ and $\Omega '$, respectively. Suppose that there exists an intertwiner $\varLambda$ such that for all $f \in {{\mathcal {D}}}(L), \varLambda f \in {{\mathcal {D}}}({{\mathscr {L}}})$,

96 $$\begin{aligned} {{\mathscr {L}}}\varLambda f= & {} \varLambda L f, \end{aligned}$$

and a duality function $D: {\hat{\Omega }} \times \Omega \rightarrow {\mathbb {R}}$ for ${\hat{L}}$ and *L*, namely $D(\xi ,\cdot ) \in {{\mathcal {D}}}(L)$ for all $\xi \in {\hat{\Omega }}, D(\cdot ,\eta ) \in {{\mathcal {D}}}({\hat{L}})$ for all $\eta \in \Omega$ and

97 $$\begin{aligned} {\hat{L}}_D= & {} L_\mathrm{right}D. \end{aligned}$$

Then, if $\varLambda _\mathrm{right}D(\xi ,\cdot ) \in {{\mathcal {D}}}({{\mathscr {L}}})$ for all $\xi \in {\hat{\Omega }}$ and $\varLambda _\mathrm{right}D(\cdot ,\zeta ) \in {{\mathcal {D}}}({\hat{L}})$ for all $\zeta \in \Omega ', \varLambda _\mathrm{right}D$ is a duality function for ${\hat{L}}$ and ${{\mathscr {L}}}$, i.e.

98 $$\begin{aligned} {\hat{L}}_{} {\varLambda }_{\mathrm{right}} D = {{{\mathscr {L}}}}_{\mathrm{right}} {\varLambda }_{\mathrm{right}} D. \end{aligned}$$

#### Proof

99 $$\begin{aligned} {\hat{L}}_{} {\varLambda }_{\mathrm{right}D} = {\varLambda }_{\mathrm{right}} {\hat{L}}_{} D = {\varLambda }_{\mathrm{right}} {L}_{\mathrm{right}} D = {{{\mathscr {L}}}}_{\mathrm{right}} {\varLambda }_{\mathrm{right}} D. \end{aligned}$$

Here in the first equality we used that left and right actions commute, in the second equality we used the assumed duality of $\hat{L}$ and *L*, and in the third equality we used the assumed intertwining. $\square$

### Intertwining Between Continuum and Discrete Processes

In this section we prove the existence of an intertwining relation between the interacting diffusion processes presented in Sect. 2.5 and the particle systems of Sect. 2.3. This intertwining relation provides a second connection, besides the scaling limit procedure (cf. Sect. 2.5), between continuum and discrete processes, which proves to be better suited for the goal of establishing duality relations among these processes. Indeed, the characterization of all possible simple factorized self-dualities for particle systems obtained in Sect. 4 and the intertwining relation below, via the application of Theorem 5.1, produces a characterization of all possible dualities, resp. self-dualities, between the discrete and the continuum processes, resp. of the continuum process.

In the following proposition, we prove the intertwining relation for operators $L^{\sigma ,\beta }$ and ${{\mathscr {L}}}^{\sigma ,\beta }$ defined, respectively, on functions $f : E^V \rightarrow {\mathbb {R}}, E= {\mathbb {N}}$, as

100 $$\begin{aligned} L^{\sigma ,\beta } f(\eta )= & {} \sum _{x,y \in V} p(x,y)\ L^{\sigma ,\beta }_{x,y} f(\eta ),\quad \eta \in E^V, \end{aligned}$$

where

101 $$\begin{aligned} L^{\sigma ,\beta }_{x,y}f(\eta )= & {} \eta _x(\beta +\sigma \eta _y) (f(\eta ^{x,y})-f(\eta )) + \eta _y (\beta +\sigma \eta _x) (f(\eta ^{y,x})-f(\eta )), \end{aligned}$$

and, on real analytic functions $f : {\mathbb {R}}^V \rightarrow {\mathbb {R}}$, as

102 $$\begin{aligned} {{\mathscr {L}}}^{\sigma ,\beta } f(z)= & {} \sum _{x,y \in V} p(x,y)\ {{\mathscr {L}}}^{\sigma ,\beta }_{x,y}f(z),\quad z \in {\mathbb {R}}^V, \end{aligned}$$

where

103 $$\begin{aligned} {{\mathscr {L}}}^{\sigma ,\beta }_{x,y}f(z)= & {} (-\beta (z_x-z_y)(\partial _x-\partial _y) +\sigma z_x z_y (\partial _x-\partial _y)^2 )f(z). \end{aligned}$$

Note that $L^{\sigma ,\beta }$ in (100) is a special instance of the generator $L^{u,v}$ in (6) with conditions (9), while ${{\mathscr {L}}}^{\sigma ,\beta }$ above, matches on a common sub-domain, for particular choices of the parameters $\sigma$ and $\beta$, those in (16).

#### Proposition 5.1

Let *G* be the Poisson probability kernel defined as the operator that maps functions $f : {\mathbb {N}}\rightarrow {\mathbb {R}}$ into functions $G f : {\mathbb {R}}\rightarrow {\mathbb {R}}$ as

104 $$\begin{aligned} G f(z)= & {} \sum _{n =0}^\infty f(n) \frac{z^n}{n!} e^{-z},\quad z \in {\mathbb {R}}. \end{aligned}$$

Then, whenever $G f : {\mathbb {R}}\rightarrow {\mathbb {R}}$ is a real analytic function, if $G^\otimes = \otimes _{x \in V} G^{(x)}$ denotes the tensorized operator mapping functions $f : {\mathbb {N}}^V \rightarrow {\mathbb {R}}$ into functions $f : {\mathbb {R}}^V \rightarrow {\mathbb {R}}$ accordingly, ${{\mathscr {L}}}^{\sigma ,\beta }$ and $L^{\sigma ,\beta }$ are intertwined with intertwiner $G^\otimes$, namely

105 $$\begin{aligned} {{\mathscr {L}}}^{\sigma ,\beta } G^\otimes f(z)= & {} G^\otimes L^{\sigma ,\beta } f(z),\quad z \in \mathbb {\mathbb {R}}^V. \end{aligned}$$

#### Proof

Let us introduce the non-normalized operator

106 $$\begin{aligned} {\bar{G}} f(z)= & {} \sum _{n=0}^\infty f(n) \frac{z^n}{n!},\quad z \in {\mathbb {R}}, \end{aligned}$$

and the associated tensorized operator ${\bar{G}}^\otimes =\otimes _{x \in V} {\bar{G}}^{(x)}$. Due to the factorized structure of $L^{\sigma ,\beta }, {{\mathscr {L}}}^{\sigma ,\beta }$ and ${\bar{G}}^\otimes$, the proof of the intertwining relation (105) with ${\bar{G}}^\otimes$ as an intertwiner reduces to consider and combine the following relations:

107 $$\begin{aligned} \sum _{n=0}^\infty n f(n-1) \frac{z^n}{n!}= & {} z {\bar{G}}f(z) \end{aligned}$$

108 $$\begin{aligned} \sum _{n=0}^\infty f(n+1) \frac{z^n}{n!}= & {} \frac{d}{dz} {\bar{G}}f(z) \end{aligned}$$

109 $$\begin{aligned} \sum _{n=0}^\infty n f(n) \frac{z^n}{n!}= & {} z \frac{d}{dz} {\bar{G}}f(z) \end{aligned}$$

110 $$\begin{aligned} \sum _{n=0}^\infty n f(n+1) \frac{z^n}{n!}= & {} z \frac{d^2}{dz^2} {\bar{G}}f(z). \end{aligned}$$

As a first consequence, we have

111 $$\begin{aligned} {{\mathscr {L}}}^{\sigma ,\beta } {\bar{G}}^\otimes f(z)= & {} {\bar{G}}^\otimes L^{\sigma ,\beta } f(z),\quad z \in {\mathbb {R}}^V. \end{aligned}$$

We obtain (105) by observing that ($|z|=\sum _{x \in V} z_x$)

112 $$\begin{aligned} G^\otimes f(z)= & {} e^{-|z|}\ {\bar{G}}^\otimes f(z),\quad z \in {\mathbb {R}}^V\ , \end{aligned}$$

and that, for $g(z) = {\bar{g}}(z)\cdot e^{-|z|}$,

113 $$\begin{aligned} {{\mathscr {L}}}^{\sigma ,\beta } g(z)= & {} e^{-|z|}\ {{\mathscr {L}}}^{\sigma ,\beta } {\bar{g}}(z),\quad z \in {\mathbb {R}}^V. \end{aligned}$$

$$\square $$

We note that the intertwiner $G^\otimes$ has a nice probabilistic interpretation: from an “energy” configuration $z \in {\mathbb {R}}_+^V$, the associated particle configurations are generated by placing a number of particles on each site $x \in V$, independently, and distributed according to a Poisson random variable with intensity $z_x$.

In the remaining part of this section, under some reasonable regularity assumptions, we are able to invert the intertwining relation (105), namely to find an operator $H^\otimes =\otimes _{x \in V} H^{(x)}$ that intertwines $L^{\sigma ,\beta }$ and ${{\mathscr {L}}}^{\sigma ,\beta }$, in this order. The natural candidate for *H* is the “inverse operator” of *G*, whenever this is well-defined. In general, this “inverse intertwiner” lacks any probabilistic interpretation, but indeed establishes a second intertwining relation useful in the next section.

#### Proposition 5.2

Let *H* be the differential operator mapping real analytic functions $g : {\mathbb {R}}\rightarrow {\mathbb {R}}$ into functions $H g : {\mathbb {N}}\rightarrow {\mathbb {R}}$ as

114 $$\begin{aligned} H g(n)= & {} \left( \left[ \frac{d^n}{d z^n} \right] _{z=0} e^z g(z) \right) ,\quad n \in {\mathbb {N}}. \end{aligned}$$

Then *H* is the inverse operator of *G*, namely, for all $f : {\mathbb {N}}\rightarrow {\mathbb {R}}$ such that $G f : {\mathbb {R}}\rightarrow {\mathbb {R}}$ is real analytic, we have

115 $$\begin{aligned} G H g (z) = g(z),\quad z \in {\mathbb {R}},\quad H G f(n) = f(n)\ ,\quad n \in {\mathbb {N}}. \end{aligned}$$

Moreover, the tensorized operator $H^\otimes =\otimes _{x \in V} H^{(x)}$ is an intertwiner for $L^{\sigma , \beta }$ and ${{\mathscr {L}}}^{\sigma ,\beta }$, i.e. for all real analytic $g : {\mathbb {R}}^V \rightarrow {\mathbb {R}}$,

116 $$\begin{aligned} L^{\sigma ,\beta } H^\otimes g(n)= & {} H^\otimes {{\mathscr {L}}}^{\sigma ,\beta } g(n),\quad n \in {\mathbb {N}}. \end{aligned}$$

Before giving the proof, we need the following lemma.

#### Lemma 1

Let *A* be the operator acting on functions $f : {\mathbb {N}}\rightarrow {\mathbb {R}}$ defined as

117 $$\begin{aligned} A f(n)= & {} \sum _{k=0}^n \left( {\begin{array}{c}n\\ k\end{array}}\right) f(k),\quad n \in {\mathbb {N}}. \end{aligned}$$

Then, the tensorized operator $A^\otimes = \otimes _{x \in V} A^{(x)}$ is a symmetry for the generator $L^{\sigma ,\beta }$, i.e. for all $f\ : {\mathbb {N}}^V \rightarrow {\mathbb {R}}$

118 $$\begin{aligned} A^\otimes L^{\sigma , \beta } f(n)= & {} L^{\sigma ,\beta } A^\otimes f(n),\quad n \in {\mathbb {N}}^V. \end{aligned}$$

#### Proof

Instead of going through tedious computations, we exploit the fact that the operator $A^\otimes$ has the form

119 $$\begin{aligned} A^\otimes= & {} \otimes _{x \in V}\ A^{(x)} = \otimes _{x \in V}\ e^{J^{(x)}} = \otimes _{x \in V}\ \sum _{k=0}^\infty \frac{(J^{(x)})^k}{k!}, \end{aligned}$$

where $J^{(x)}$ is an operator defined for functions $f : {\mathbb {N}}^V \rightarrow {\mathbb {R}}$ which acts only on the *x*th variable as

120 $$\begin{aligned} J^{(x)} f(n)= & {} n_x\ f(n-\delta _x),\quad n \in {\mathbb {N}}^V. \end{aligned}$$

Since all these operators $\{J^{(x)},\ x \in V \}$ commute over the sites we have

121 $$\begin{aligned} A^\otimes= & {} \otimes _{x \in V} e^{J^{(x)}} = e^{\sum _{x \in V} J^{(x)}}. \end{aligned}$$

We conclude the proof by noting that the operator

122 $$\begin{aligned} \sum _{x \in V} J^{(x)} \end{aligned}$$

is a symmetry for the generator $L^{\sigma , \beta }$, cf. e.g. [1, 9]. $\square$

#### Proof

(Proposition 5.2) First we compute the following key relations:

123 $$\begin{aligned} \left( \left[ \frac{d^n}{dz^n} \right] _{z=0} g'(z)\right)= & {} \left( \left[ \frac{d^{n+1}}{dz^{n+1}} \right] _{z=0} g(z)\right) \end{aligned}$$

124 $$\begin{aligned} \left( \left[ \frac{d^n}{dz^n} \right] _{z=0} z g(z)\right)= & {} n \left( \left[ \frac{d^{n-1}}{dz^{n-1}} \right] _{z=0} g(z)\right) \end{aligned}$$

125 $$\begin{aligned} \left( \left[ \frac{d^n}{dz^n} \right] _{z=0} z g'(z) \right)= & {} n \left( \left[ \frac{d^n}{dz^n} \right] _{z=0} g(z)\right) \end{aligned}$$

126 $$\begin{aligned} \left( \left[ \frac{d^n}{dz^n} \right] _{z=0} z g''(z) \right)= & {} n \left( \left[ \frac{d^{n+1}}{dz^{n+1}} \right] _{z=0} g(z) \right) . \end{aligned}$$

Hence, if we introduce the operator

127 $$\begin{aligned} {\bar{H}} g(n) = \left( \left[ \frac{d^n}{d z^n} \right] _{z=0} g(z) \right) ,\quad n \in {\mathbb {N}}, \end{aligned}$$

and the associated tensorized operator ${\bar{H}}^\otimes = \otimes _{x \in V} {\bar{H}}^{(x)}$, we obtain

128 $$\begin{aligned} L^{\sigma ,\beta } {\bar{H}}^\otimes g(n) = {\bar{H}}^\otimes {{\mathscr {L}}}^{\sigma ,\beta } g(n),\quad n \in {\mathbb {N}}. \end{aligned}$$

Now, by using Lemma 1 and noting that

129 $$\begin{aligned} H g(n) = \sum _{k=0}^n \left( {\begin{array}{c}n\\ k\end{array}}\right) {\bar{H}} g(k) = A {\bar{H}} g(n),\quad n \in {\mathbb {N}}, \end{aligned}$$

and, by the mixed property of the tensor product,

130 $$\begin{aligned} H^\otimes g(n) = (A {\bar{H}})^\otimes g(n) = A^\otimes {\bar{H}}^\otimes g(n),\quad n \in {\mathbb {N}}^V, \end{aligned}$$

we get (116) by applying first (130), then (118) and finally (128):

131 $$\begin{aligned} L^{\sigma ,\beta } H^\otimes g = L^{\sigma ,\beta } A^\otimes {\bar{H}}^\otimes g = A^\otimes L^{\sigma ,\beta } {\bar{H}}^\otimes g = A^\otimes {\bar{H}}^\otimes {{\mathscr {L}}}^{\sigma ,\beta } g = H^\otimes {{\mathscr {L}}}^{\sigma ,\beta } g. \end{aligned}$$

$$\square $$

### Generating Functions and Duality

As anticipated in the previous section, from the intertwining relation (105) and the functions obtained in Sect. 4.1, in what follows we find explicitly new duality relations.

Due to the factorized form (4) of the self-duality functions with single-site functions (61) and (67) and the tensor form of the intertwiner $G^\otimes$ in (105), the new functions inherit the same factorized form. Moreover, from the definition of *G* in (104), the whole computation reduces to determine *(exponential) generating functions* of (61) and (67). To this purpose, some identities for hypergeometric functions are available, cf. e.g. the tables in [13, Chap. 9]. Moreover, all generating functions obtained satisfy the requirements of analyticity for suitable choices of the parameters $\sigma$, $\beta$, *a* and *b* (cf. [13]), hence all operations below make sense.

However, just as the functions found in Sect. 4.1, the functions here obtained will only be “candidate” (self-)duality functions, since no duality relation as in (1) has been proved, yet. By using the “inverse” intertwining (116), all these “possible” dualities turn out to be equivalent, i.e. one implies all the others. Thus, in Proposition 5.3, we choose to prove directly the self-duality relation for the continuum process, more immediate to verify due to the simpler form of the self-duality functions. Indeed, while the single-site self-duality functions for the $\text {SIP}(\alpha )$ process, for instance, have the generic form of an hypergeometric function132the single-site duality functions between discrete and continuum processes involve in their expressions hypergeometric functions133and those for the self-duality of continuum processes are even simpler, namely134as the number of arguments of the hypergeometric function drops.

The tables below schematically report all single-site (self)-duality functions for the operators $L^{\sigma ,\beta }$ in (100) and ${{\mathscr {L}}}^{\sigma ,\beta }$ in (102). Recall that the parameters *a*, $b \in {\mathbb {R}}$ in (9) are properly chosen (cf. Sect. 4.1).

(I) *Independent random walkers* ($\text {IRW}$), $$\sigma =0\ $$, $$\beta =1\ $$.

**Table:** 

Classical polynomials	$\frac{n!}{(n-k)!} b^k{\mathbb {1}}\{k \le n\}$	$\left( bz\right) ^k$	$e^{- v} e^{ b v z}$
Orthogonal polynomials	$a^k C_k(n;-\frac{a}{b})$	$\left( a + b z \right) ^k$	$e^{(a-1)v}e^{b v z}$
Cheap duality functions	$e^{\lambda } \frac{k!}{\lambda ^k}{\mathbb {1}}\{k=n \}$	$e^{\lambda -z}\left( \frac{z}{\lambda } \right) ^k$	$e^{\lambda -z-v}e^{ \frac{v z}{\lambda }}$

(II) *Symmetric inclusion process* ($\text {SIP}(\alpha )$), $$\sigma =1\ $$, $$\beta =\alpha > 0\ $$.

**Table:** 

Cl.	$\frac{n!}{(n-k)!} \frac{\Gamma \left( \alpha \right) }{\Gamma \left( \alpha + k\right) } \left( b \alpha \right) ^k {\mathbb {1}}\{k \le n\}$	$\frac{\Gamma (\alpha )}{\Gamma (\alpha +k)} \left( b \alpha z \right) ^k$	
Or.	$a^k \frac{\Gamma \left( \alpha \right) }{\Gamma \left( \alpha + k \right) } M_k(n; \frac{a}{a-b\alpha },\alpha )$		
Ch.	$(1-\lambda )^\alpha \frac{k!}{\lambda ^k} \frac{\Gamma (\alpha )}{\Gamma (\alpha +k)} 1\{k=n \}$	$(1-\lambda )^\alpha e^{-z} \left( \frac{z}{\lambda }\right) ^k \frac{\Gamma (\alpha )}{\Gamma (\alpha +k)}$

(III) *Symmetric exclusion process* ($\text {SEP}(\gamma )$) , $$\sigma =-1\ $$, $$\beta =\gamma \in {\mathbb {N}}\ $$.

**Table:** 

Cl.			
Or.			
Ch.		$(1+\lambda )^{-\gamma }e^{-z}\left( \frac{z}{\lambda }\right) ^k \tfrac{\gamma !}{(\gamma -k)!}$	$e^{-z-v} \left( \frac{\lambda +v z}{\lambda (1+\lambda )}\right) ^{\gamma }$

More in detail, on the left-most column we place the single-site self-duality functions *d*(*k*, *n*) for the particle systems of Sect. 2.3: while the top-left functions are those already appearing in [1, 9], cf. also Sect. 2.3 and (66)—and, thus, for this reason denoted here as the “classical” ones - the second-to-the-top functions are those derived in Sect. 4.1 in (61)–(67) and being related to suitable families of orthogonal polynomials. While these two classes of single-site self-duality functions satisfy condition (5) (they are the only ones doing so), the bottom-left single-site self-duality functions correspond to the “cheap” self-duality (cf. end of Sect. 2.2), namely the detailed-balance condition w.r.t. the measures $\{\otimes _{x \in V} \nu _\lambda ,\ \lambda > 0 \}$ with marginals (8).

On the mid-column, we find the single-site duality functions between the difference operators $L^{\sigma ,\beta }$ and the differential operators ${{\mathscr {L}}}^{\sigma ,\beta }$, obtained from their left-neighbors by a direct application of the operator *G* in (104) on the *n*-variables. The new functions will depend hence on the two variables $k \in {\mathbb {N}}$ and $z \in {\mathbb {R}}_+$.

A second application w.r.t. the *k*-variables of the same operator *G* on the functions just obtained gives us back the right-most column, functions depending now on variables $v, z \in {\mathbb {R}}_+$. These functions represent the single-site self-duality functions for the differential operator ${{\mathscr {L}}}^{\sigma ,\beta }$. As an immediate consequence of Proposition 5.2, we could also proceed from right to left by applying the inverse intertwiner *H* in (114).

Note that the single-site self-duality functions for ${{\mathscr {L}}}^{\sigma ,\beta }$ on the right-most columns, though they have been derived from different discrete analogues, i.e. classical, orthogonal and cheap single-site functions, within the same table they differ only for a factor which depends only on the conserved quantities $|z|=\sum _{x \in V}z_x$ and $|v|=\sum _{x \in V}v_x$. Henceforth, when proving the self-duality relation, this extra-factor does not play any role and it is enough to check that the functions135for constants $c \in {\mathbb {R}}$, are single-site self-duality functions for the operators ${{\mathscr {L}}}^{0,1}$, ${{\mathscr {L}}}^{1,\alpha }$ and ${{\mathscr {L}}}^{-1,\gamma }$, respectively. This final computation is the content of the next proposition.

#### Proposition 5.3

For any constant $c \in {\mathbb {R}}$, the functions *d*(*v*, *z*) in (135) are single-site self-duality functions for the differential operators ${{\mathscr {L}}}^{0,1}, {{\mathscr {L}}}^{1,\alpha }$ and ${{\mathscr {L}}}^{-1,\gamma }$, respectively.

#### Proof

To prove that

136 $$\begin{aligned} d(v,z)= & {} e^{c v z},\quad v, z \in {\mathbb {R}}_+, \end{aligned}$$

is a single-site self-duality function for the differential operator ${{\mathscr {L}}}^{0,1}$, we first observe that

137 $$\begin{aligned} \partial _{z} d(v,z)= & {} c v\ d(v,z). \end{aligned}$$

Hence, the self-duality relation for the single-edge generator ${{\mathscr {L}}}^{1,0}_{x,y}$ rewrites

138 $$\begin{aligned}&-(v_x - v_y)(cz_x - cz_y) d(v_x, z_x) d(v_y, z_y) \nonumber \\&\quad = - (z_x - z_y) (cv_x -cv_y) d(v_x, z_x) d(v_y, z_y), \end{aligned}$$

which indeed holds.

For the second proof of self-duality for the single-site function139we use the following shortcut: for $x \in V$,140Additionally, we recall a formula for the $z_x$-derivative of $F_x$, namely

141 $$\begin{aligned} \frac{\partial }{\partial z_x} F_x(\alpha )= \frac{c v_x}{\alpha } F_x(\alpha +1), \end{aligned}$$

and a recurrence identity

142 $$\begin{aligned} F_x(\alpha +1) = F_x(\alpha ) - \frac{c v_x z_x}{\alpha (\alpha +1)} F_x(\alpha +2). \end{aligned}$$

Hence, the self-duality relation for ${{\mathscr {L}}}^{1,\alpha }_{x,y}$ w.r.t. the function $F_x(\alpha ) F_y(\alpha )$ rewrites by using (141) as

143 $$\begin{aligned}&c z_x v_y F_x(\alpha +1) F_y(\alpha ) + c v_x z_y F_x(\alpha ) F_y(\alpha +1) \nonumber \\&\quad \quad + \frac{c^2 (v_x z_x)(v_y z_x)}{\alpha (\alpha +1)} F_x(\alpha +2) F_y(\alpha ) + \frac{c^2 (v_x z_y)(v_y z_y)}{\alpha (\alpha +1)} F_x(\alpha )F_y(\alpha +2) \nonumber \\&\quad =c v_x z_y F_x(\alpha +1) F_y(\alpha ) + c v_y z_x F_x(\alpha ) F_y(\alpha +1)\nonumber \\&\quad \quad + \frac{c^2 (v_x z_x) (v_x z_y)}{\alpha (\alpha +1)} F_x(\alpha +2) F_y(\alpha ) + \frac{c^2 (z_x v_y)(z_y v_y)}{\alpha (\alpha +1)} F_x(\alpha )F_y(\alpha +2). \end{aligned}$$

By substituting (142), the identity holds.

The proof for the operator ${{\mathscr {L}}}^{-1,\gamma }$ follows the same lines and we omit it. $\square$
